# Integrated Design of Materials and Structures for Flexible Base Asphalt Pavement

**DOI:** 10.3390/ma18153602

**Published:** 2025-07-31

**Authors:** Bin Huang, Qinxue Pan, Xiaolong Chen, Jia Hu, Songtao Lv

**Affiliations:** 1 School of Transportation, Changsha University of Science and Technology, Changsha 410114, China; 18736249418@163.com (B.H.); cxl74253@163.com (X.C.); hj0805hj@163.com (J.H.); lst@csust.edu.cn (S.L.); 2 National Key Laboratory of Green and Long-Life Road Engineering in Extreme Environment, Changsha 410114, China; 3 National Engineering Research Center of Highway Maintenance Technology, Changsha 410114, China; 4 Hunan Provincial Expressway Group Co., Ltd., Changsha 410153, China

**Keywords:** asphalt pavement, structural design, bi-modulus, mechanical response analysis, integration

## Abstract

Current asphalt pavement structural design methods often lack a strong quantitative link to materials’ mixtures and mechanical properties and typically ignore the significant tensile–compressive disparities of materials, resulting in notable analysis errors. This study employed the dual-modulus theory to numerically analyze flexible base asphalt pavements under varied configurations, revealing how critical structural responses and fatigue life evolve. This examination also determined optimal layer mixes through mechanical parameter modeling for integrated material–structure design. The results showed that fundamental responses and fatigue life vary nonlinearly with thickness and modulus. The effect of modulus outweighed that of thickness, with the effects of the tensile modulus being more pronounced than compressive ones, and surface transverse strain being most sensitive to both. The recommended compressive–tensile modulus ratios were about 1.5, 2.0, and 1.2 for upper, lower, and base layers, respectively. By using this integrated design method, the optimized pavement structures achieved superior stress distribution, significantly extending the base service life. As a result, more realistic design lifetimes were obtained.

## 1. Introduction

The combined effects of traffic loads and environmental factors cause fatigue cracking and rutting in asphalt pavements. These phenomena significantly compromise pavements’ load-bearing capacity and durability. Traditional design methods often suffer from mismatching layer performance and improper thickness combinations, with poorly controlled flexible base stiffness and strength acting as key bottlenecks in improving structural performance. To enhance the overall structural performance, it is imperative to analyze correlations between design parameters and materials’ mechanical properties, overcome the limitations of material–structure separation, and establish an integrated optimization framework based on fundamental mechanics to improve service performance throughout the entire lifecycle of the pavement [1,2,3].

The selection of mechanical parameters for asphalt pavement materials is critical for ensuring the reliability of structural calculations and design [4,5,6,7,8]. However, current pavement structural design practices primarily rely on engineering experience or specification-recommended ranges for selected mechanical parameters, with each parameter chosen independently. The synergistic effects of material composition and external environmental changes on the parameters are not systematically considered, resulting in a lack of scientific reliability to determine the parameters. Moreover, the significant difference in mechanical properties of pavement materials under tension and compression leads to the material having non-unique mechanical parameters [9]. In addition, due to the separation of pavement structure, material mix design, and mechanical parameter determination, there is a deviation of greater than 50% between the pavement structure design results and the actual pavement performance. This difference makes it difficult for current pavement structure design standards to play a controlling role. Therefore, it is of great significance to integrate and analyze asphalt pavement materials and structures by considering the differences in mechanical properties between tension and compression of materials [10,11].

Scientifically sound and rational structural design can significantly enhance a pavement’s service performance and extend its operational lifespan [12,13]. Various studies have shown [14,15,16] that the thickness and modulus values of individual pavement structural layers have a substantial influence on the critical mechanical responses of the pavement system. Pavement performance is optimized when the thicknesses of asphalt surface layers and the flexible base course are maintained in the ranges of 18–20 cm and 35–40 cm, respectively. Increasing the modulus of structural layers effectively reduces surface deflection, thereby enhancing load-bearing capacity and decreasing surface distress rates. A reduction in the compressive-to-tensile modulus ratio of pavement materials significantly decreases overall structural stiffness and markedly alters mechanical responses across layers. In addition, internationally adopted durable pavement designs often extend service life by increasing the thickness or stiffness of fatigue-resistant layers. While these studies address the effects of structural layer thickness and modulus on pavement performance, they have failed to conduct a systematic investigation into the quantitative relationship between material properties and structural mechanical behavior, as well as their implications for integrated pavement design.

Recent advancements have been made in the integrated design of pavement materials and structure [17,18,19,20]. For example, in the United States, the concept of integrated design is reflected in perpetual asphalt pavement systems, where high-performance surface materials are selected and combined with mechanical analysis of the pavement structure [21]. In China, researchers have proposed composition-based design methods for pavement materials and structure, derived from structural calculations and experimental studies focused on pavement performance requirements [22,23]. However, these integrated approaches lack standardization. Most studies select materials based on functional requirements of the pavement structure, focusing either on structural mechanical performance analysis or on how different mix designs affect the pavement mixture’s service properties, such as high-temperature stability, low-temperature cracking resistance, and moisture susceptibility. Few investigations have explored the critical linkage between material mechanical properties and structural mechanical verification design. Relevant studies have clearly shown [24,25] that the mechanical properties of asphalt pavement materials are significantly influenced by factors such as binder content and air void ratio, and changes in these parameters directly affect the mechanical behavior and service life of the pavement structure. Therefore, it is necessary to develop integrated material–structure design methodologies based on mechanical property compatibility.

According to classical linear elastic theory, materials exhibit identical elastic behavior under tension and compression. However, numerous studies [26,27,28] have demonstrated that materials possess significant tension–compression asymmetry in their mechanical parameters, resulting in substantial deviations between calculated mechanical responses and actual field performance. Zhang et al. [29] questioned the rationality of using a single modulus in conventional rigid pavement design and, using bi-modulus theory, found that the maximum tensile stress at critical locations was approximately 10% lower than that predicted by traditional methods. Cheng et al. [30] developed a method to determine the compressive and tensile moduli of asphalt mixtures through indirect tensile testing, revealing distinct differences in the dynamic compressive–tensile modulus and Poisson’s ratio of AC-13 mixtures under varying temperatures and loading frequencies. Lv et al. [31] identified significant differences between the tensile and compressive moduli of asphalt mixtures, suggesting that both parameters should not be treated equivalently in pavement structural design. Pan et al. [32] established a finite element method based on bi-modulus theory for asphalt pavement analysis, and comparative results indicated that deviations between bi-modulus and traditional linear elastic models could reach as high as 50%. Therefore, the significant tension–compression asymmetry of materials must be accounted for in the integrated design of asphalt pavement materials and structure.

In summary, this study combines a quantitative model of the change in tensile and compressive mechanical parameters for typical flexible base asphalt pavement materials, considering both internal and external influencing factors. Additionally, it conducts a structural analysis of flexible base asphalt pavements under various thickness and material parameter combinations, based on the bi-modulus theory. It also reveals the change law of the key mechanical response and fatigue life with influencing factors, establishing a corresponding quantification model. Based on these results, the asphalt pavement structural design is optimized, and combined with its mechanical parameter quantification model, to back-calculate the mix proportion of the structural layer. This methodology effectively resolves the problem of separation between structural design and material proportion design. And it preliminarily realized the integrated design of asphalt pavement materials and structure. The research results can provide a reference for the structural design of asphalt durable pavement.

## 2. Multi-Factor Coupled Quantitative Model of Tensile and Compressive Mechanical Parameters of Pavement Materials

Based on previous experimental studies of material mechanical properties conducted by the author’s research team [33,34,35,36,37,38,39,40], and in compliance with the relevant provisions of JTG E20-2019 [41] and JTG E51-2024 [42], this study adopted uniaxial compression tests and direct tension tests to determine the compressive and tensile moduli and Poisson’s ratios of asphalt pavement materials. The obtained mechanical parameters were then used to select appropriate modulus values for each structural layer of the pavement, followed by necessary corrections to the material modulus calculations. This systematic approach ensures accurate characterization of pavement material properties for subsequent analysis.

Asphalt mixtures exhibit pronounced viscoelastic characteristics, with their mechanical parameters being significantly influenced by temperature, binder content, loading rate, and air void percentage. To explore the nonlinear characteristics of the tensile and compressive mechanical parameters of asphalt mixture, the tensile and compressive moduli and Poisson’s ratios of asphalt mixture were tested by a uniaxial compression test and a direct tension test, considering the influence of factors such as temperature (5 °C, 15 °C, 20 °C, 30 °C, 40 °C), binder content (3.5%, 4%, 4.5%, 5%, 5.5%), loading rate (0.01 MPa/s, 0.02 MPa/s, 0.05 MPa/s, 0.1 MPa/s, 0.5 MPa/s), and void ratio (4.2%, 8.3%, 12.6%, 16.3%, 19.7%).

The analytical results demonstrate that both the compressive and tensile moduli of asphalt mixture decrease as the temperature increases and gradually stabilize after exceeding 40 °C. Firstly, the compressive and tensile Poisson’s ratios slowly increase, and then, with the sharp increase in temperature, the compressive Poisson’s ratio may even exceed 0.5 when the temperature is greater than 30 °C. With the increase in asphalt dosage, the compressive and tensile moduli of the asphalt mixture first increase and then decrease. The ranges of the compressive and tensile moduli are within 30% and 22%, respectively. The compressive and tensile moduli peak at the asphalt dosage of 4.75%. The compressive and tensile Poisson’s ratios decrease first and then increase with the increase in asphalt dosage, and the ranges of the compressive and tensile Poisson’s ratios are within 3.5% and 10%, respectively. The compressive and tensile moduli increase with the increase in the loading rate, showing a power function relationship. As the loading rate continues to increase, the increase in the compressive and tensile moduli gradually decreases. The compressive and tensile Poisson’s ratios decrease as the loading rate increases, and then the decrease rate gradually slows down. With the increase in the void ratio, both the compressive and tensile moduli exhibit a downward trend (the reduction can reach 58%). The compressive and tensile Poisson’s ratios increase with the increase in the void ratio, and the maximum increase in compressive and tensile Poisson’s ratios exceeds 70%. Based on this, a quantitative model of the change in the tensile and compressive mechanical parameters of asphalt mixture with multiple factors, such as loading rate, temperature, binder dosage, and void ratio, was established as shown in Equations (1)–(4). The correlation coefficient (R^2^) is superior to 0.94. Hence, the model accuracy is high.

(1) $$E^{c}=\sqrt{\left[4213+3452/{\left(0.0019+V_{c}/4512\right)}^{2}\right]\times 1.5^{-0.42C^{2}+4.21C-12.51-0.0066CT-0.21T}}v^{-0.0014T+0.0036{V_{c}}^{2}-0.069V_{c}+0.49}$$

(2) $$E^{t}=\sqrt{\left[1849+1321/{\left(0.0016+V_{c}/2351\right)}^{2}\right]\times 1.2^{-0.63C^{2}+6.41C-13.19-0.015CT-0.56T}}v^{-0.0015T+0.0027{V_{c}}^{2}-0.052V_{c}+0.56}$$

(3) $$\mu^{c}=\left(0.025V_{c}+0.36\right)\times \left[0.49+\frac{1.02\times 10^{0.05T}}{45.35+10^{0.05}}+\left(0.0025T-0.069C^{2}-0.66C-1.64\right)\times 10^{-10v}\right]$$

(4) $$\mu^{t}=\left(0.0027V^{c}+0.0032\right)\times \left[35.28+\frac{27.37\times 10^{0.05T}}{24.32+10^{0.05T}}+\left(0.049T-0.81C^{2}+7.55C-13.93\right)\times 10^{-10v}\right]$$

where *E^c^*—uniaxial compressive modulus (MPa); *E^t^*—direct tensile modulus (MPa); *μ^c^*—uniaxial compressive Poisson’s ratio; *μ^t^*—direct tensile Poisson’s ratio; *C*—binder content (%); *v*—loading rate (MPa/s); *T*—temperature (°C); and *Vc*—void ratio (%)

## 3. Calculation Method of Asphalt Pavement Structure Based on Bi-Modulus Theory

### 3.1. Fundamental Characteristics of Bi-Modulus Theory

#### 3.1.1. Fundamental Assumptions

**Figure 1:**
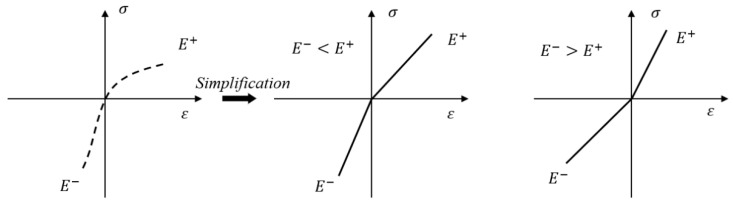
Schematic diagram of bilinear constitutive model.

Figure 1 illustrates that the constitutive model of bi-modulus materials demonstrates nonlinear behavior, where the stress–strain relationship can be approximated as bilinear with two distinct slopes [29].

Bi-modulus materials satisfy the following fundamental assumptions [29]. The material is homogeneous and isotropic, exhibiting distinct elastic properties depending on the sign of the principal stress. The material demonstrates small-deformation behavior. Under the principal stress sign criterion, both the elastic modulus and Poisson’s ratio vary with the sign (tension–compression) of the principal stress. The relationship between moduli and Poisson’s ratios satisfies *E*^+^/*μ*^+^ = *E*^−^/*μ*^−^.

#### 3.1.2. Constitutive Model

The principal stress discrimination rule determines the material’s mechanical properties according to the sign and direction of the principal stresses. Its constitutive relations are governed by the relationship between principal stresses and principal strains, and the stress–strain relationship under the principal stress discrimination rule is as follows:

(5) $$\left\{\begin{array}{l} \varepsilon_{\alpha }\\ \varepsilon_{\beta }\\ \varepsilon_{\gamma } \end{array}\right\}=A\left\{\begin{array}{l} \sigma_{\alpha }\\ \sigma_{\beta }\\ \sigma_{\gamma } \end{array}\right\},A=\left[\begin{array}{l} \;\frac{1}{E^{\alpha }}\;\;\;-\frac{\mu^{\beta }}{E^{\beta }}\;\;-\frac{\mu^{\gamma }}{E^{\gamma }}\\ -\frac{\mu^{\alpha }}{E^{\alpha }}\;\;\;\frac{1}{E^{\beta }}\;\;\;-\frac{\mu^{\gamma }}{E^{\gamma }}\\ -\frac{\mu^{\alpha }}{E^{\alpha }}\;\;-\frac{\mu^{\beta }}{E^{\beta }}\;\;\;\frac{1}{E^{\gamma }} \end{array}\right]$$

where *ε*_1_, *ε*_2_, *ε*_3_ are principal strains; *σ*_1_, *σ*_2_, *σ*_3_ are principal stresses. *A* is the compliance matrix. *μ^α^*, *μ^α^*, *μ^γ^* are Poisson’s ratios in principal stress directions. *E^α^*, *E^α^*, *E^γ^* are moduli in principal stress directions. Elastic parameters are assigned based on the nature of principal stresses: the tensile modulus (*E*^+^) and tensile Poisson’s ratio (*μ*^+^) under tension, and the compressive modulus (*E^−^*) and compressive Poisson’s ratio under compression (*μ^−^*).

The general constitutive equation in an arbitrary direction can be derived by using the conversion formula between stress and strain in different coordinates, as shown in Equations (6)–(12).

(6) $$\begin{array}{l} \varepsilon_{x}\;=[(\frac{l_{1}^{2}}{2G_{\alpha }}+A_{\alpha })l_{1}^{2}+(\frac{m_{1}^{2}}{2G_{\beta }}+A_{\beta })m_{1}^{2}+(\frac{n_{1}^{2}}{2G_{\gamma }}+A_{\gamma })n_{1}^{2}]\sigma_{x}+2[(\frac{l_{1}^{2}}{2G_{\alpha }}+A_{\alpha })l_{1}l_{2}+(\frac{m_{1}^{2}}{2G_{\beta }}+A_{\beta })m_{1}m_{2}+(\frac{n_{1}^{2}}{2G_{\gamma }}+A_{\gamma })n_{1}n_{2}]\tau_{xy}\\ \;\;\;+[(\frac{l_{1}^{2}}{2G_{\alpha }}+A_{\alpha })l_{2}^{2}+(\frac{m_{1}^{2}}{2G_{\beta }}+A_{\beta })m_{2}^{2}+(\frac{n_{1}^{2}}{2G_{\gamma }}+A_{\gamma })n_{2}^{2}]\sigma_{y}+2[(\frac{l_{1}^{2}}{2G_{\alpha }}+A_{\alpha })l_{2}l_{3}+(\frac{m_{1}^{2}}{2G_{\beta }}+A_{\beta })m_{2}m_{3}+(\frac{n_{1}^{2}}{2G_{\gamma }}+A_{\gamma })n_{2}n_{3}]\tau_{yz}\\ \;\;\;+[(\frac{l_{1}^{2}}{2G_{\alpha }}+A_{\alpha })l_{3}^{2}+(\frac{m_{1}^{2}}{2G_{\beta }}+A_{\beta })m_{3}^{2}+(\frac{n_{1}^{2}}{2G_{\gamma }}+A_{\gamma })n_{3}^{2}]\sigma_{z}+2[(\frac{l_{1}^{2}}{2G_{\alpha }}+A_{\alpha })l_{1}l_{3}+(\frac{m_{1}^{2}}{2G_{\beta }}+A_{\beta })m_{1}m_{3}+(\frac{n_{1}^{2}}{2G_{\gamma }}+A_{\gamma })n_{1}n_{3}]\tau_{xz} \end{array}$$

(7) $$\begin{array}{l} \varepsilon_{y}\;=[(\frac{l_{2}^{2}}{2G_{\alpha }}+A_{\alpha })l_{1}^{2}+(\frac{m_{2}^{2}}{2G_{\beta }}+A_{\beta })m_{1}^{2}+(\frac{n_{2}^{2}}{2G_{\gamma }}+A_{\gamma })n_{1}^{2}]\sigma_{x}+2[(\frac{l_{2}^{2}}{2G_{\alpha }}+A_{\alpha })l_{1}l_{2}+(\frac{m_{2}^{2}}{2G_{\beta }}+A_{\beta })m_{1}m_{2}+(\frac{n_{2}^{2}}{2G_{\gamma }}+A_{\gamma })n_{1}n_{2}]\tau_{xy}\\ \;\;\;+[(\frac{l_{2}^{2}}{2G_{\alpha }}+A_{\alpha })l_{2}^{2}+(\frac{m_{2}^{2}}{2G_{\beta }}+A_{\beta })m_{2}^{2}+(\frac{n_{2}^{2}}{2G_{\gamma }}+A_{\gamma })n_{2}^{2}]\sigma_{y}+2[(\frac{l_{2}^{2}}{2G_{\alpha }}+A_{\alpha })l_{2}l_{3}+(\frac{m_{2}^{2}}{2G_{\beta }}+A_{\beta })m_{2}m_{3}+(\frac{n_{2}^{2}}{2G_{\gamma }}+A_{\gamma })n_{2}n_{3}]\tau_{yz}\\ \;\;\;+[(\frac{l_{2}^{2}}{2G_{\alpha }}+A_{\alpha })l_{3}^{2}+(\frac{m_{2}^{2}}{2G_{\beta }}+A_{\beta })m_{3}^{2}+(\frac{n_{2}^{2}}{2G_{\gamma }}+A_{\gamma })n_{3}^{2}]\sigma_{z}+2[(\frac{l_{2}^{2}}{2G_{\alpha }}+A_{\alpha })l_{1}l_{3}+(\frac{m_{2}^{2}}{2G_{\beta }}+A_{\beta })m_{1}m_{3}+(\frac{n_{2}^{2}}{2G_{\gamma }}+A_{\gamma })n_{1}n_{3}]\tau_{xz} \end{array}$$

(8) $$\begin{array}{l} \varepsilon_{z}\;=[(\frac{l_{3}^{2}}{2G_{\alpha }}+A_{\alpha })l_{1}^{2}+(\frac{m_{3}^{2}}{2G_{\beta }}+A_{\beta })m_{1}^{2}+(\frac{n_{3}^{2}}{2G_{\gamma }}+A_{\gamma })n_{1}^{2}]\sigma_{x}+2[(\frac{l_{3}^{2}}{2G_{\alpha }}+A_{\alpha })l_{1}l_{2}+(\frac{m_{3}^{2}}{2G_{\beta }}+A_{\beta })m_{1}m_{2}+(\frac{n_{3}^{2}}{2G_{\gamma }}+A_{\gamma })n_{1}n_{2}]\tau_{xy}\\ \;\;\;+[(\frac{l_{3}^{2}}{2G_{\alpha }}+A_{\alpha })l_{2}^{2}+(\frac{m_{3}^{2}}{2G_{\beta }}+A_{\beta })m_{2}^{2}+(\frac{n_{3}^{2}}{2G_{\gamma }}+A_{\gamma })n_{2}^{2}]\sigma_{y}+2[(\frac{l_{3}^{2}}{2G_{\alpha }}+A_{\alpha })l_{2}l_{3}+(\frac{m_{3}^{2}}{2G_{\beta }}+A_{\beta })m_{2}m_{3}+(\frac{n_{3}^{2}}{2G_{\gamma }}+A_{\gamma })n_{2}n_{3}]\tau_{yz}\\ \;\;\;+[(\frac{l_{3}^{2}}{2G_{\alpha }}+A_{\alpha })l_{3}^{2}+(\frac{m_{3}^{2}}{2G_{\beta }}+A_{\beta })m_{3}^{2}+(\frac{n_{3}^{2}}{2G_{\gamma }}+A_{\gamma })n_{3}^{2}]\sigma_{z}+2[(\frac{l_{3}^{2}}{2G_{\alpha }}+A_{\alpha })l_{1}l_{3}+(\frac{m_{3}^{2}}{2G_{\beta }}+A_{\beta })m_{1}m_{3}+(\frac{n_{3}^{2}}{2G_{\gamma }}+A_{\gamma })n_{1}n_{3}]\tau_{xz} \end{array}$$

(9) $$\begin{array}{l} \gamma_{xy}=[\frac{l_{1}l_{2}}{G_{\alpha }}l_{1}^{2}+\frac{m_{1}m_{2}}{G_{\beta }}m_{1}^{2}+\frac{n_{1}n_{2}}{G_{\gamma }}n_{1}^{2}]\sigma_{x}+[\frac{l_{1}l_{2}}{G_{\alpha }}l_{2}^{2}+\frac{m_{1}m_{2}}{G_{\beta }}m_{2}^{2}+\frac{n_{1}n_{2}}{G_{\gamma }}n_{2}^{2}]\sigma_{y}\\ \;\;\;\;\;\;+[\frac{l_{1}l_{2}}{G_{\alpha }}l_{3}^{2}+\frac{m_{1}m_{2}}{G_{\beta }}m_{3}^{2}+\frac{n_{1}n_{2}}{G_{\gamma }}n_{3}^{2}]\sigma_{z}+[\frac{l_{1}l_{2}}{G_{\alpha }}2l_{1}l_{2}+\frac{m_{1}m_{2}}{G_{\beta }}2m_{1}m_{2}+\frac{n_{1}n_{2}}{G_{\gamma }}2n_{1}n_{2}]\tau_{xy}\\ \;\;\;\;\;\;+[\frac{l_{1}l_{2}}{G_{\alpha }}2l_{1}l_{3}+\frac{m_{1}m_{2}}{G_{\beta }}2m_{1}m_{3}+\frac{n_{1}n_{2}}{G_{\gamma }}2n_{1}n_{3}]\tau_{xz}+[\frac{l_{1}l_{2}}{G_{\alpha }}2l_{2}l_{3}+\frac{m_{1}m_{2}}{G_{\beta }}2m_{2}m_{3}+\frac{n_{1}n_{2}}{G_{\gamma }}2n_{2}n_{3}]\tau_{yz} \end{array}$$

(10) $$\begin{array}{l} \gamma_{xz}=[\frac{l_{1}l_{3}}{G_{\alpha }}l_{1}^{2}+\frac{m_{1}m_{3}}{G_{\beta }}m_{1}^{2}+\frac{n_{1}n_{3}}{G_{\gamma }}n_{1}^{2}]\sigma_{x}+[\frac{l_{1}l_{3}}{G_{\alpha }}l_{2}^{2}+\frac{m_{1}m_{3}}{G_{\beta }}m_{2}^{2}+\frac{n_{1}n_{3}}{G_{\gamma }}n_{2}^{2}]\sigma_{y}\\ \;\;\;\;\;\;+[\frac{l_{1}l_{3}}{G_{\alpha }}l_{3}^{2}+\frac{m_{1}m_{3}}{G_{\beta }}m_{3}^{2}+\frac{n_{1}n_{3}}{G_{\gamma }}n_{3}^{2}]\sigma_{z}+[\frac{l_{1}l_{3}}{G_{\alpha }}2l_{1}l_{2}+\frac{m_{1}m_{3}}{G_{\beta }}2m_{1}m_{2}+\frac{n_{1}n_{3}}{G_{\gamma }}2n_{1}n_{2}]\tau_{xy}\\ \;\;\;\;\;\;+[\frac{l_{1}l_{3}}{G_{\alpha }}2l_{1}l_{3}+\frac{m_{1}m_{3}}{G_{\beta }}2m_{1}m_{3}+\frac{n_{1}n_{3}}{G_{\gamma }}2n_{1}n_{3}]\tau_{xz}+[\frac{l_{1}l_{3}}{G_{\alpha }}2l_{2}l_{3}+\frac{m_{1}m_{3}}{G_{\beta }}2m_{2}m_{3}+\frac{n_{1}n_{3}}{G_{\gamma }}2n_{2}n_{3}]\tau_{yz} \end{array}$$

(11) $$\begin{array}{l} \gamma_{yz}=[\frac{l_{2}l_{3}}{G_{\alpha }}l_{1}^{2}+\frac{m_{2}m_{3}}{G_{\beta }}m_{1}^{2}+\frac{n_{2}n_{3}}{G_{\gamma }}n_{1}^{2}]\sigma_{x}+[\frac{l_{2}l_{3}}{G_{\alpha }}l_{2}^{2}+\frac{m_{2}m_{3}}{G_{\beta }}m_{2}^{2}+\frac{n_{2}n_{3}}{G_{\gamma }}n_{2}^{2}]\sigma_{y}\\ \;\;\;\;\;\;+[\frac{l_{2}l_{3}}{G_{\alpha }}l_{3}^{2}+\frac{m_{2}m_{3}}{G_{\beta }}m_{3}^{2}+\frac{n_{2}n_{3}}{G_{\gamma }}n_{3}^{2}]\sigma_{z}+[\frac{l_{2}l_{3}}{G_{\alpha }}2l_{1}l_{2}+\frac{m_{2}m_{3}}{G_{\beta }}2m_{1}m_{2}+\frac{n_{2}n_{3}}{G_{\gamma }}2n_{1}n_{2}]\tau_{xy}\\ \;\;\;\;\;\;+[\frac{l_{2}l_{3}}{G_{\alpha }}2l_{1}l_{3}+\frac{m_{2}m_{3}}{G_{\beta }}2m_{1}m_{3}+\frac{n_{2}n_{3}}{G_{\gamma }}2n_{1}n_{3}]\tau_{xz}+[\frac{l_{2}l_{3}}{G_{\alpha }}2l_{2}l_{3}+\frac{m_{2}m_{3}}{G_{\beta }}2m_{2}m_{3}+\frac{n_{2}n_{3}}{G_{\gamma }}2n_{2}n_{3}]\tau_{yz} \end{array}$$

where

(12) $$\left.\begin{array}{l} G_{\alpha }=E^{\alpha }/\left[2(1+\mu^{\alpha }\right)],A_{\alpha }=-\mu^{\alpha }/E^{\alpha }\\ G_{\beta }=E^{\beta }/\left[2(1+\mu^{\beta }\right)],A_{\beta }=-\mu^{\beta }/E^{\beta }\\ G_{\gamma }=E^{\gamma }/\left[2(1+\mu^{\gamma })\right],A_{\gamma }=-\mu^{\gamma }/E^{\gamma } \end{array}\right\}$$

where *l_i_*, *m_i_*, and *n_i_* (*i* = 1, 2, 3) are direction cosines between the arbitrary direction coordinate axes and the principal direction coordinate axes, respectively.

### 3.2. Numerical Calculation Method of Bi-Modulus Theory

#### 3.2.1. Finite Element Format

The element iteration format of the finite element calculation under classical linear elastic theory is formulated as follows:

(13) $$K^{e}U^{e}=F_{f}^{e}+F_{s}^{e}$$

where *K^e^* is the element stiffness matrix; *U^e^* is the element nodal displacement vector; *F^e^_f_* is the body force of the element; and *F^e^_s_* is the surface force of the element.

Subsequently, the overall stiffness matrix *K* is obtained through the assembly of all element stiffness matrices.

*Ku* = *F* (14)

where *Ku* is the displacement matrix of all nodes of the structure and *F* is the external load matrix of all nodes of the structure.

The primary distinction between the bi-modulus theory and the finite element method based on classical linear elasticity theory lies in the elasticity matrix *D*. The other steps are the same as the finite element method of classical linear elasticity. The elasticity matrix *D* of the bi-modulus theory is derived from Equation (15).

(15) $$D=\bar{L_{\varepsilon }^{T}}D_{I}{\bar{L}}_{\varepsilon }$$

*D_I_* = *A* (16)

(17) $$\bar{L_{\varepsilon }}=\left[\begin{array}{cccccc} l_{1}^{2} & m_{1}^{2} & n_{1}^{2} & m_{1}n_{1} & n_{1}l_{1} & l_{1}m_{1}\\ l_{2}^{2} & m_{2}^{2} & n_{2}^{2} & m_{2}n_{2} & n_{2}l_{2} & l_{2}m_{2}\\ l_{3}^{2} & m_{3}^{2} & n_{3}^{2} & m_{3}n_{3} & n_{3}l_{3} & l_{3}m_{3}\\ 2l_{1}l_{2} & 2m_{1}m_{2} & 2n_{1}n_{2} & m_{1}n_{2}+n_{1}m_{2} & n_{1}l_{2}+l_{1}n_{2} & l_{1}m_{2}+l_{2}m_{1}\\ 2l_{2}l_{3} & 2m_{2}m_{3} & 2n_{2}n_{3} & m_{2}n_{3}+n_{2}m_{3} & n_{2}l_{3}+l_{2}n_{3} & l_{2}m_{3}+l_{3}m_{2}\\ 2l_{3}l_{1} & 2m_{3}m_{1} & 2n_{3}n_{1} & m_{3}n_{1}+n_{3}m_{1} & n_{3}l_{1}+l_{3}n_{1} & l_{3}m_{1}+l_{1}m_{3} \end{array}\right]$$

By substituting the elasticity matrix *D* into Equation (18), the stiffness matrix can be determined. Subsequent procedures follow the classical linear elastic finite element method.

(18) $$K=\int_{V}B^{T}DBdv$$

where $D_{I}$ is the elasticity matrix in the principal directions. *L_ε_* is the transformation matrix. *B* is the strain–displacement matrix. *B* is the strain–displacement matrix.

#### 3.2.2. Numerical Calculation Method

The bi-modulus theory is a special nonlinear problem that occurs after the bilinear simplification of the material’s constitutive relationship. It can be solved by iterative technology, and its iterative format is as follows:

*K*_*i*−1_*u_i_* = *F* (19)

where *K_i_*_−1_ is the global stiffness matrix computed at iteration step *i*−1 and *μ_i_* is the nodal displacement vector obtained from the solution at iteration step *i*.

The specific calculation steps are as follows:

Step 1: Assuming that the material modulus is the single modulus, the initial elastic parameters of the structure are assigned by the total tensile state or total compressive state, and the initial elastic matrix *D*^+^ or *D*^−^ is obtained. Then, the stress and strain of each element are calculated.

Step 2: Determine the magnitude and direction of principal stress in Gaussian integral points of each element and judge the positive and negative of its principal stress, thus obtaining the flexibility matrix *A* in the direction of the principal stress at each integration point. Then, the stiffness matrix *D* of bi-modulus theory is obtained by using Equation (15).

Step 3: The stress and strain of each element are calculated according to the new stiffness matrix.

Step 4: Discrimination: Calculate the displacement difference of each node or the stress deviation in each element’s Gaussian integral point between the ith and i-1th iteration. If the control criterion requirements are met, the computation is complete. Otherwise, go to step 2 and start the next iterative computation.

**Figure 2:**
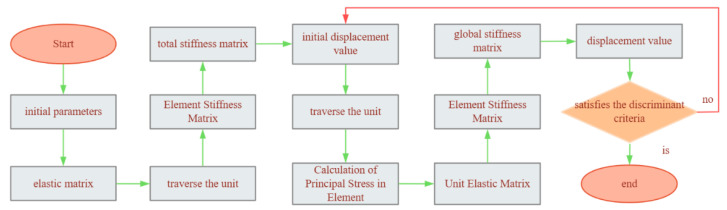
Computational flowchart of the bi-modulus theory finite element method.

Figure 2 shows the computational flowchart for the bi-modulus theory-based finite element method.

### 3.3. Establishment and Verification of Numerical Model

**Figure 3:**
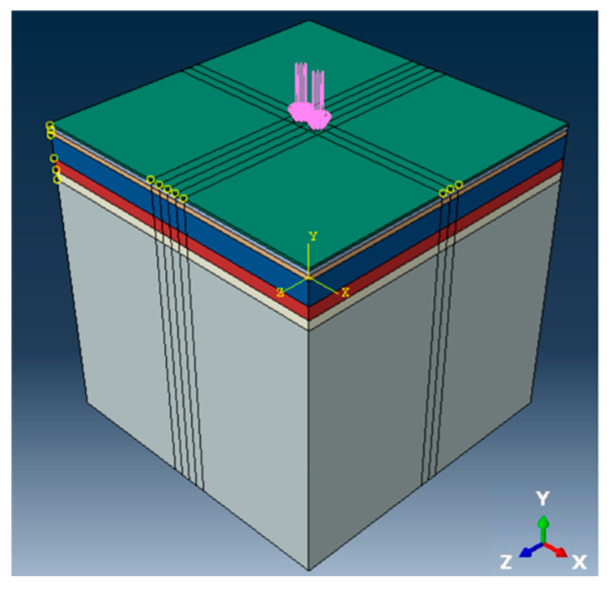
Three-dimensional numerical model of pavement structure.

Based on the above calculation method, a three-dimensional bi-modulus pavement structure calculation subroutine was developed through the ABAQUS 2021 secondary development platform (UMAT). And according to the JTG D50-2017 [43] in China, Figure 3 shows the finite element model of flexible base asphalt pavement under standard load.

The numerical model of the pavement structure adopts a dual-circular vertical uniformly distributed load model based on the elastic layered continuous system. The radius of a single circular load (σ) was 10.65 cm, and the center-to-center spacing between the dual circular loads was 31.95 cm. In the diagram, the x, y, and z axes represent the transverse direction (pavement width), vertical direction (pavement depth), and longitudinal direction (traffic direction), respectively. The element type was C3D8 (an 8-node linear brick element). The model assumes fully bonded interlayer conditions between pavement layers. The model dimensions were 6 m × 6 m × 6 m, with a uniformly distributed load of 0.7 MPa. Boundary conditions included zero normal displacement on all lateral sides and fully fixed constraints at the model base. The mesh in the load application area was refined, with element sizes gradually increasing from the center of the load outward. The maximum element size did not exceed 0.02 m × 0.02 m. The model consisted of 327,712 elements and 342,111 nodes.

**Figure 4:**
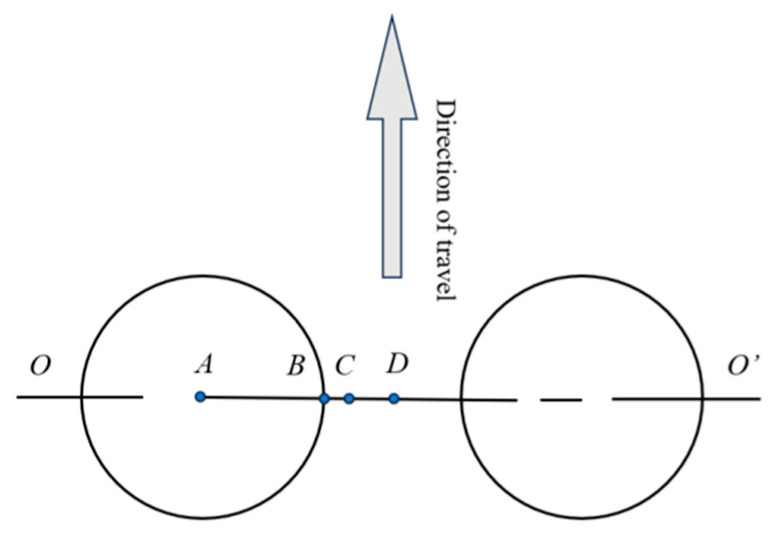
Schematic diagram of critical analysis points.

Figure 4 illustrates the critical analysis points. Point A is the wheel load center (center of the loaded area); Point B is the transverse wheel load edge point (lateral edge of the loaded area); Point D is the wheel gap center (midpoint between dual wheel gaps); and Point C is the midpoint line between Points B and D, forming a transverse line passing through the wheel load center and wheel gap center.

**Figure 5:**
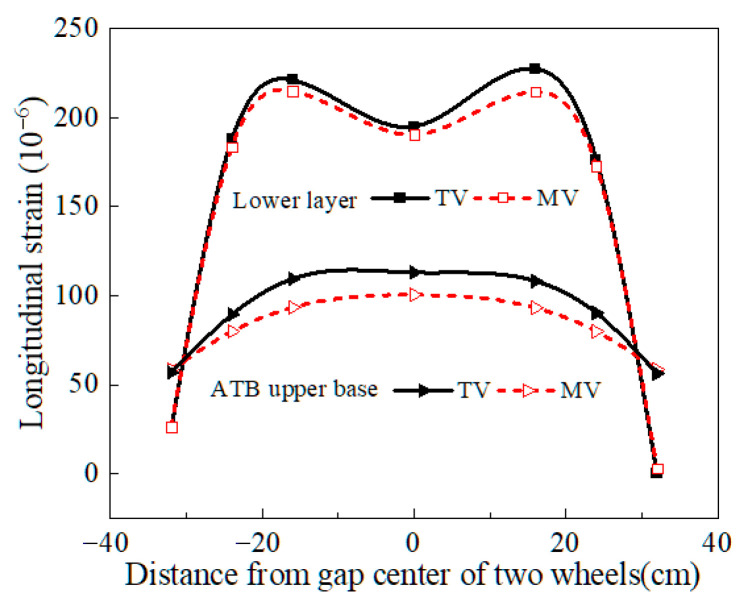
Comparison between measured and theoretical values.

**Figure 6:**
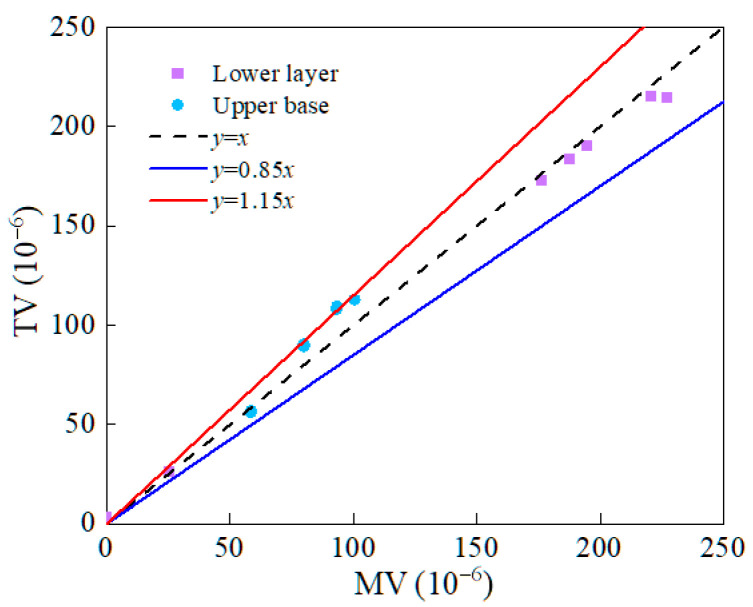
Strain error analysis.

To verify the reliability of the asphalt pavement numerical model, a typical flexible base asphalt pavement was selected as a case study. Theoretical values (TVs) and measured values (MVs) of longitudinal and transverse strains in both the surface and base layers were compared. Figure 5 and Figure 6 show the results of this test.

Figure 5 and Figure 6 show that the TV and MV of longitudinal and transverse strains in both the surface and base layers exhibit similar trends. But there are certain differences in the values. The errors between the TV and MV, calculated using the bi-modulus theory, are generally within 15%, which shows that the established numerical model is reasonable and reliable.

### 3.4. Pavement Structure Calculation and Analysis Program

The study investigated the influence of varying thicknesses and modulus ratios of the surface, base, and subbase layers on the mechanical response of the typical flexible base asphalt pavement structure. Based on existing pavement design standards [20,43] and the authors’ previous research [44], the compressive-to-tensile modulus ratio of asphalt materials generally ranges from 1 to 2. Accordingly, Table 1 shows the selection of mechanical parameters and thicknesses of each structural layer. When analyzing the influence of a specific factor on mechanical response, other design parameters remain fixed. Given that modified asphalt is typically used for the upper and middle surface layers in high-grade highways, the mechanical parameters of these layers are assigned identical values throughout the analysis.

## 4. Analysis of Key Mechanical Responses of Asphalt Pavement

### 4.1. Deflection

**Figure 7:**
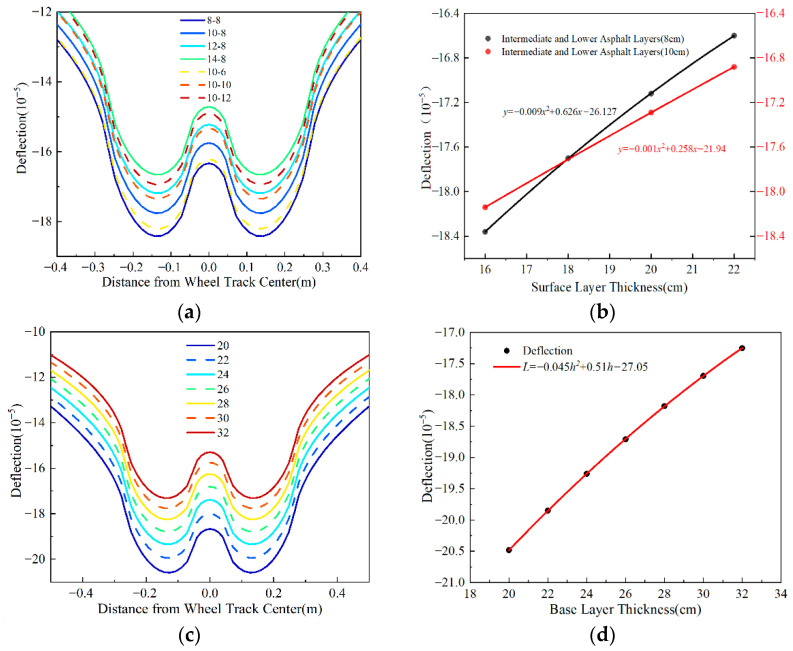
Variation patterns of pavement surface deflection with thickness. (**a**) Deflection under different surface layer thicknesses. (**b**) Relationship between maximum deflection and thickness. (**c**) Deflection under different base layer thicknesses. (**d**) Maximum deflection and thickness relationship.

In pavement structural analysis, pavement surface deflection was commonly adopted to characterize the structural stiffness of the entire pavement system. This study examined the variation patterns of surface deflection under different influencing factors, with a focus on adjusting the thickness of the upper and intermediate layers (10 cm) and the lower layer (8 cm), while keeping other parameters constant. ${oo}^{\prime }$ consistent methodology was applied when analyzing other influencing factors. The representative path (designated as Line ${oo}^{\prime }$ in Figure 4) was selected for mechanical analysis, and Figure 7 presents the deflection calculation results illustrating the variation patterns under these factors.

Figure 7 shows that the maximum deflection decreases as the surface layer thickness increases. When thickening the upper, intermediate, and lower layers by 6 cm, the maximum deflection decreases by 10%, 10%, and 7%, respectively. This phenomenon indicates that the deflection sensitivity of the upper and intermediate layers exceeds that of the lower layer. Deflection also diminishes with increasing base layer thickness, and the amplitude of deflection variation reduces progressively as the base layer thickens. The relationship between maximum deflection and base layer thickness follows a quadratic function. Specifically, a 10 cm increase in base layer thickness reduces the maximum deflection by approximately 15%. This scenario demonstrates that flexible base asphalt pavement base layer thickness significantly impacts deflection values. Enhancing base layer thickness effectively reduces surface deflection, thereby improving the pavement’s load-bearing capacity.

**Figure 8:**
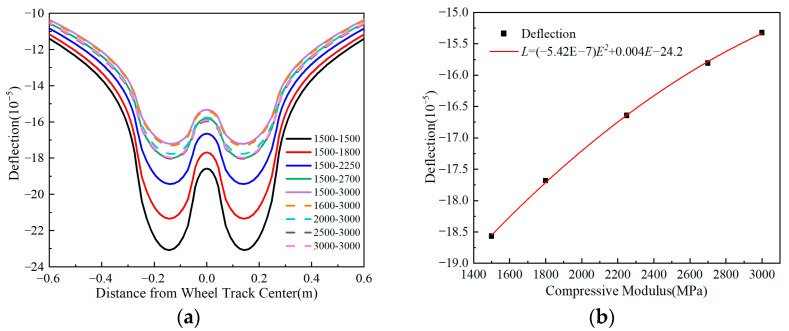
Deflection under surface layer modulus ratios. (**a**) Deflection under different surface layer modulus ratios. (**b**) Relationship between maximum deflection and modulus ratio.

Figure 8 depicts that the deflection decreases as the compressive modulus of the surface layer increases, regardless of the tensile modulus. Conversely, deflection increases as the tensile modulus increases, regardless of the compressive modulus. When increasing both tensile and compressive moduli by 900 MPa, the deflection values change by approximately 12% and 3%, respectively. These results indicate that the compressive modulus has a more significant impact on the deflection performance of the pavement structure compared to the tensile modulus, which exerts a relatively minor influence. Further analysis reveals a quadratic function relationship between the maximum surface deflection and the compressive modulus, with the deflection variation amplitude diminishing as the compressive modulus increases. In conclusion, this study identified the compressive modulus as the critical mechanical parameter governing deflection behavior in flexible base asphalt pavements.

**Figure 9:**
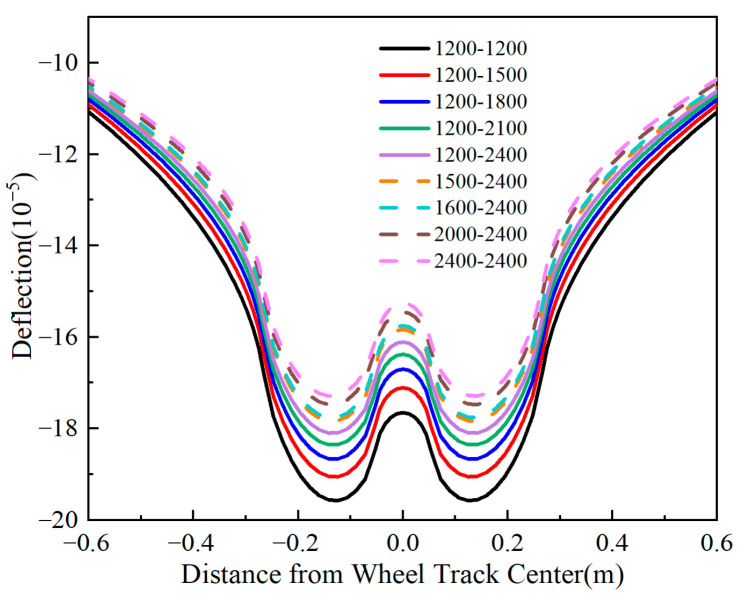
Deflection under different base layer modulus ratios.

Figure 9 demonstrates that increasing either the base layer tensile modulus or compressive modulus reduces the maximum pavement surface deflection. However, when increasing the tensile and compressive moduli by 1000 MPa, the deflection variation amplitude remains within 5%. This analysis proves that enhancing base layer stiffness can reduce deflection in flexible base asphalt pavements. Nevertheless, the magnitude of this effect is relatively limited.

### 4.2. Pavement Surface Transverse Strain

**Figure 10:**
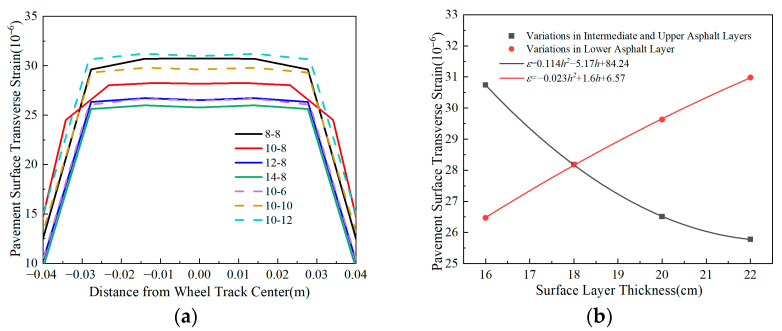
Comparison of pavement surface transverse strain with different surface layer thicknesses. (**a**) Pavement surface transverse strain. (**b**) Peak transverse strain vs. thickness.

**Figure 11:**
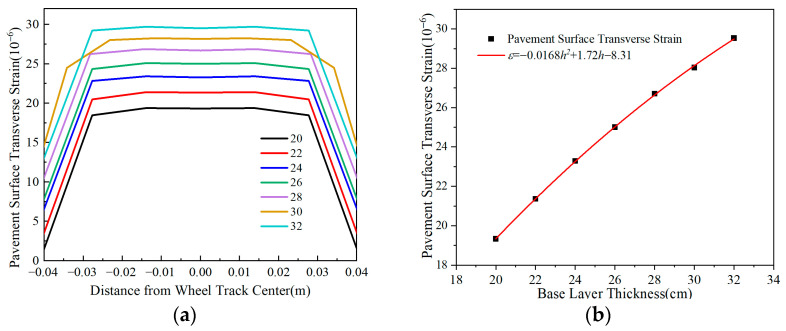
Comparison of pavement surface transverse strain i with different base layer thicknesses. (**a**) Pavement surface transverse strain. (**b**) Peak transverse strain vs. thickness.

**Figure 12:**
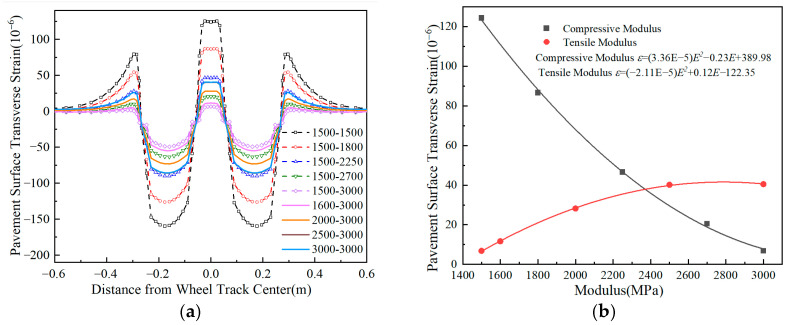
Comparison of pavement surface transverse strain with different surface layer modulus ratios. (**a**) Pavement surface transverse strain. (**b**) Peak transverse strain vs. modulus ratio.

**Figure 13:**
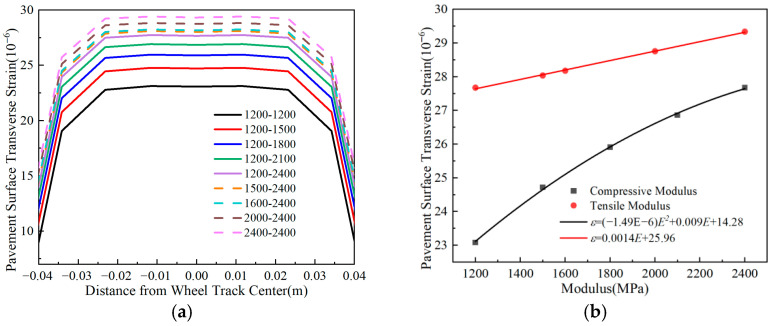
Comparison of pavement surface transverse strain with different base layer modulus ratios. (**a**) Pavement surface transverse strain. (**b**) Peak transverse strain vs. modulus ratio.

The line labeled ${oo}^{\prime }$ in Figure 4 is designated as the analysis path. Figure 10, Figure 11, Figure 12 and Figure 13 demonstrate the variation laws of pavement surface transverse stress and strain under the influences of traffic load, temperature gradient, and material properties.

Figure 10 shows that variations in surface layer thickness exert a significant impact on pavement surface transverse strain. The maximum transverse tensile strain decreases with the increase in the upper-middle surface layer thickness, while it increases with the growth of lower surface layer thickness. When increasing the thickness of the upper-middle and lower surface layers by 6 cm, the maximum transverse strain at the pavement surface changes by approximately 19% and 17%, respectively. These results indicate that the sensitivity of the pavement surface transverse strain of the upper-middle and lower surface layers is essentially identical. Appropriately increasing the thickness of the middle-upper surface layers can effectively reduce the surface-layer transverse tensile strain, thereby mitigating tensile failure in the surface layer.

Figure 11 depicts that transverse tensile strain at the pavement surface increases with base layer thickness, although the rate of increase declines as thickness grows. Specifically, a 10 cm thickness increase raises the maximum transverse tensile strain by about 45%, demonstrating that base layer thickness significantly influences surface strain behavior in flexible-base asphalt pavements. Reducing thickness effectively decreases surface strain, suggesting that strategic thickness reduction can optimize the mechanical response and stress distribution in pavement structures.

The data in Figure 13 demonstrate that the pavement surface’s transverse tensile strain decreases as the compressive modulus increases, regardless of the tensile modulus, while the opposite trend is observed when compressive modulus remains fixed. Specifically, a 1500 MPa increase in the tensile modulus amplifies maximum transverse strain by approximately fivefold, whereas an equivalent compressive modulus enhancement achieves about 95% strain reduction. These results demonstrate that surface layer moduli predominantly govern transverse strain behavior in flexible-base asphalt pavements, with concurrent compressive modulus increment and tensile modulus reduction constituting an effective strain control strategy.

Figure 11 shows that increasing either the base layer compressive modulus or tensile modulus elevates surface layer tensile strain. Specifically, a 1200 MPa increase in the compressive modulus raises the maximum transverse tensile strain by about 20%, whereas the same increase in the tensile modulus causes less than 6% strain variation. These results demonstrate that the base layer compressive modulus has a more significant impact on surface transverse tensile strain, and reducing the compressive modulus can effectively mitigate surface strain.

### 4.3. Longitudinal Stress and Strain at the Bottom of the Base Layer

**Figure 14:**
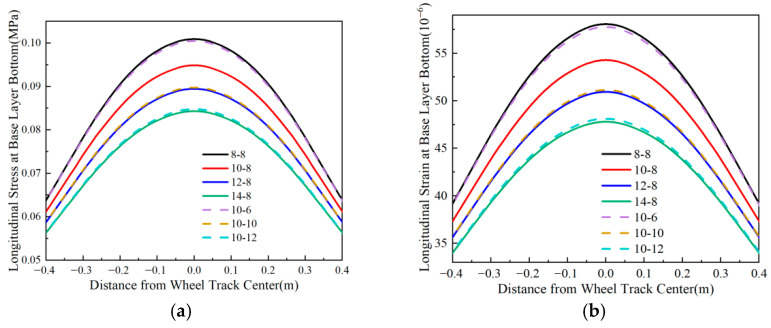
Longitudinal stress and strain at the bottom of the base layer under varying surface layer thicknesses. (**a**) Longitudinal stress at the bottom of the base layer. (**b**) Longitudinal strain at the bottom of the base layer.

**Figure 15:**
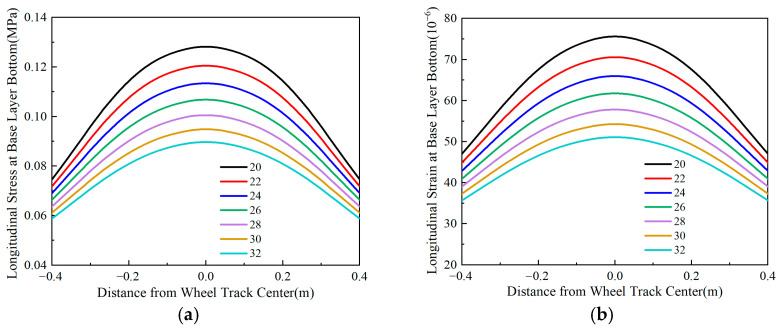
Longitudinal stress and strain at the bottom of the base layer under varying base layer thicknesses. (**a**) Longitudinal stress at the bottom of the base layer. (**b**) Longitudinal strain at the bottom of the base layer.

Using Line *oo*′ in Figure 4 as the analysis path, Figure 12, Figure 13, Figure 14 and Figure 15 illustrate the variation patterns of longitudinal stress at the bottom of the base layer under different influencing factors.

Figure 12 shows that both the longitudinal stress and longitudinal strain at the bottom of the base layer decrease as the surface layer thickness increases. The maximum values of longitudinal stress and strain exhibit a quadratic functional relationship with surface layer thickness. Adjusting the thickness of the upper-middle surface layer and lower surface layer has minimal impact on the longitudinal tensile stress at the bottom of both the base layer and subbase layer, regardless of the total surface layer thickness. When the surface layer thickness increases by 6 cm, the maximum longitudinal tensile stress and tensile strain at the bottom of the base layer decrease by approximately 20%. Therefore, increasing the surface layer thickness effectively mitigates tensile stress and strain in the base layer of flexible base asphalt pavement.

Figure 15 shows that in flexible-base asphalt pavements, both the longitudinal tensile stress and longitudinal tensile strain at the base layer bottom decrease as the base layer thickness increases. The maximum tensile stress and maximum tensile strain follow a quadratic relationship with thickness, and their reduction rates slow as thickness increases. Specifically, a 10 cm thickness increase reduces the maximum longitudinal tensile stress by 35% and the maximum longitudinal tensile strain by 39%, demonstrating that tensile strain is more sensitive to thickness changes than tensile stress. Consequently, effectively increasing base layer thickness reduces tensile stress and strain at the bottom of the base layer, leading to improved pavement durability.

**Figure 16:**
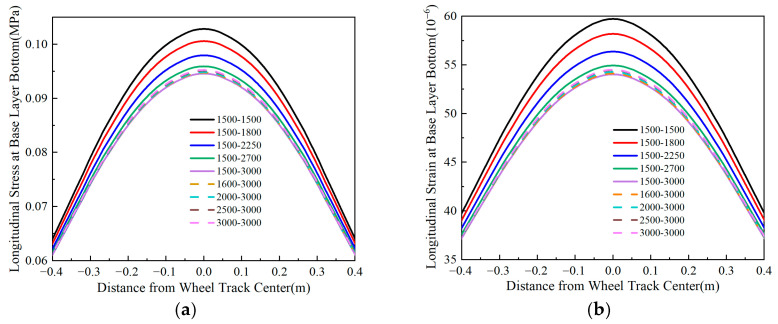
Longitudinal stress and strain at the bottom of the base layer under varying surface layer modulus ratios. (**a**) Longitudinal stress at the bottom of the base layer. (**b**) Longitudinal strain at the bottom of the base layer.

Figure 16 shows that the longitudinal tensile stress and strain at the base layer bottom of flexible-base asphalt pavements demonstrate contrasting responses to surface layer modulus variations, showing a reduction with a higher compressive modulus but an increase with a greater tensile modulus. The quantitative analysis reveals that while surface layer tensile modulus changes induce less than 1% variation in base layer responses, compressive modulus modifications cause approximately 5% change. These findings confirm that surface layer modulus adjustments have a limited influence (≤5% variation) on the mechanical behavior at the base layer bottom in this pavement system.

**Figure 17:**
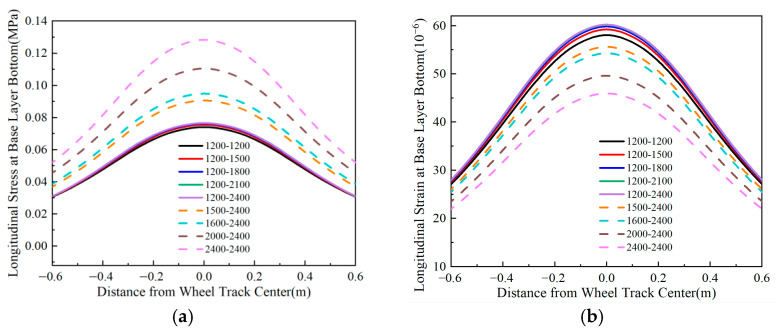
Longitudinal stress and strain at the bottom of the base layer under varying base layer modulus ratios. (**a**) Longitudinal stress at the bottom of the base layer. (**b**) Longitudinal strain at the bottom of the base layer.

Figure 17 illustrates that the base layer compressive modulus has a limited effect on tensile stress and strain regardless of the tensile modulus. Conversely, with a constant compressive modulus, increasing the tensile modulus raises base bottom tensile stress (up to 68% at +1200 MPa) while reducing tensile strain (24% at the same increment). These results establish the base layer tensile modulus as the primary control parameter for mechanical responses in flexible-base asphalt pavements, where a strategic increase can optimize strain distribution.

## 5. Integrated Design of Materials and Structures

### 5.1. Fatigue Life Analysis

This study calculated the fatigue cracking life of the base and subbase layers, as well as the fatigue life of the surface layer in the pavement structure, based on the structural verification methods described in China’s JTG D50-2017 [43].

#### 5.1.1. Fatigue Life Calculation of Asphalt Pavement Structural Layers

(1) Fatigue cracking life calculation of the asphalt surface layer, calculated according to Equation (20).

(20) $$N_{f1}=6.32\times 10^{15.96-0.29\beta }k_{a}k_{b}k_{T1}^{-1}{\left(\frac{1}{\varepsilon_{a}}\right)}^{3.97}{\left(\frac{1}{E_{a}}\right)}^{1.58}{\left(VFA\right)}^{2.72}$$

where *N_f_*_1_ is the fatigue cracking life of the asphalt mixture layer; *k_a_* is the adjustment factor for seasonal permafrost regions; and *k_b_* is the fatigue loading mode coefficient, calculated according to Equation (21).

(21) $$k_{b}={\left[\frac{1+0.3E_{a}^{0.43}{\left(VFA\right)}^{-0.85}e^{0.024h_{a}-5.41}}{1+e^{0.024h_{a}-5.41}}\right]}^{3.33}$$

where *E_a_* is the dynamic compressive modulus of the asphalt mixture at 20 °C (MPa); *VFA* is the asphalt saturation (%) (voids filled with asphalt); *h_a_* is the asphalt layer thickness; *k*_*T*1_ is the temperature adjustment coefficient; and *ε_a_* is the tensile strain at the bottom of the asphalt mixture layer (10^−6^). The dynamic modulus of the asphalt mixture was selected based on specifications and the correlations between dynamic modulus and static modulus. Table 2 shows the calculation parameters.

(2) The fatigue cracking life calculation of the inorganic binder-stabilized layer was conducted.

The fatigue cracking life of the inorganic binder-stabilized layer was calculated using Equation (22).

(22) $$N_{f2}=k_{a}k_{T2}^{-1}10^{a-b\frac{\sigma_{t}}{R_{s}}+k_{c}-0.57\beta }$$

where *N_f_*_2_ is the fatigue cracking life of the inorganic binder-stabilized layer; *k_a_* is the adjustment factor for seasonal permafrost regions; *k_T_*_2_ is the temperature adjustment coefficient; *Rs* is the flexural tensile strength of the inorganic binder-stabilized material (MPa); *a*, *b* are the regression parameters from fatigue tests; and *k_c_* is the field comprehensive correction coefficient, calculated according to Equation (23).

(23) $$k_{c}=c_{1}e^{c_{2}(h_{a}+h_{b})}+c_{3}$$

where *c*_1_, *c*_2_, and *c*_3_ are selected according to the code; *h_a_* and *h_b_* are the thicknesses of the bituminous mixture layer and the inorganic binder layer above the calculation point, respectively; *β* is the target reliability index; and *σ_t_* is the bottom tensile stress of the inorganic binder layer.

Table 3 and Table 4 show the parameters selected to calculate the service life of the inorganic binder layer according to China’s JTG D50-2017 [43].

#### 5.1.2. Variation Patterns of Pavement Structural Layer Fatigue Life with Multiple Factors

**Figure 18:**
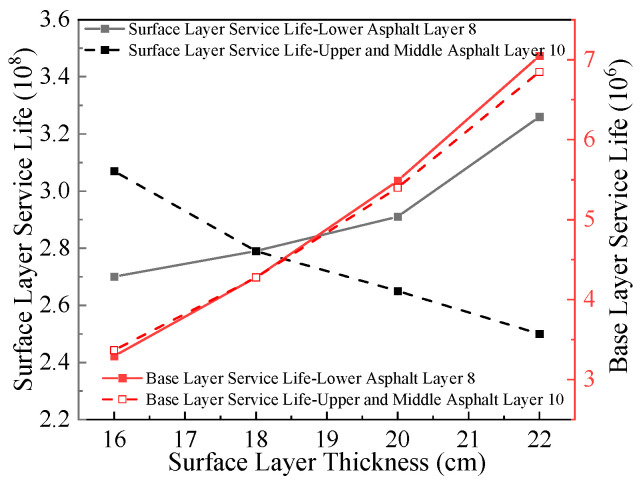
Relationship between fatigue life and surface layer thickness.

**Figure 19:**
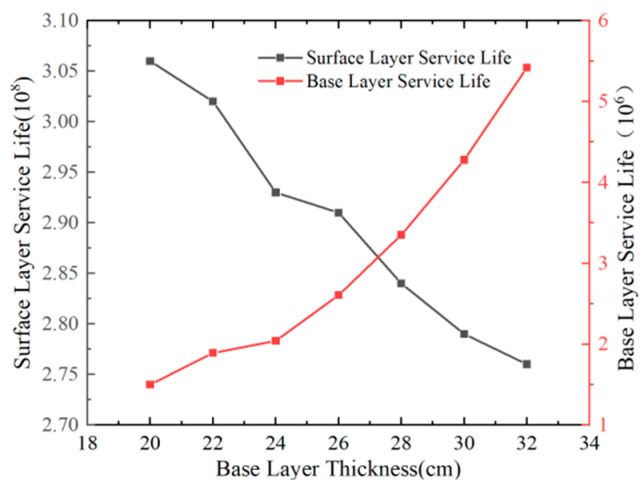
Relationship between fatigue life and base layer thickness.

Based on the aforementioned calculation methodology, Figure 16, Figure 17, Figure 18 and Figure 19 illustrate the fatigue life results of each structural layer under varying thicknesses.

Figure 16 illustrates that when the lower surface layer thickness remains constant, the fatigue life of the pavement surface layer increases with the increase in upper-middle surface layer thickness. Conversely, when the upper-middle surface layer thickness remains unchanged, the fatigue life of the pavement surface layer decreases as the lower surface layer thickness increases. A 6 cm increase in thickness results in an improvement of approximately 140% in the fatigue life of the base course. The fatigue life of the base course increases with the overall pavement surface layer thickness. However, under constant total surface layer thickness, variations in upper-middle surface layer and subbase layer thicknesses exhibit a negligible impact on the base course fatigue life. Based on the aforementioned analysis, increasing the total thickness of the pavement surface layer will significantly enhance the fatigue life of the base course.

According to Figure 19, the fatigue life of the pavement surface layer decreases with increasing base course thickness, while the fatigue life of the base course increases with its thickness. Additionally, the fatigue life of the flexible pavement surface layer significantly exceeds that of the base course. A 10 cm increase in base course thickness results in an improvement of approximately 180% in the base course fatigue life, whereas the surface layer fatigue life decreases by approximately 10%. The variation magnitude of fatigue life across structural layers indicates that base course thickness has the most pronounced impact on its own fatigue life. In pavement structural design, it is recommended to moderately increase the base course thickness to enhance its fatigue life.

**Figure 20:**
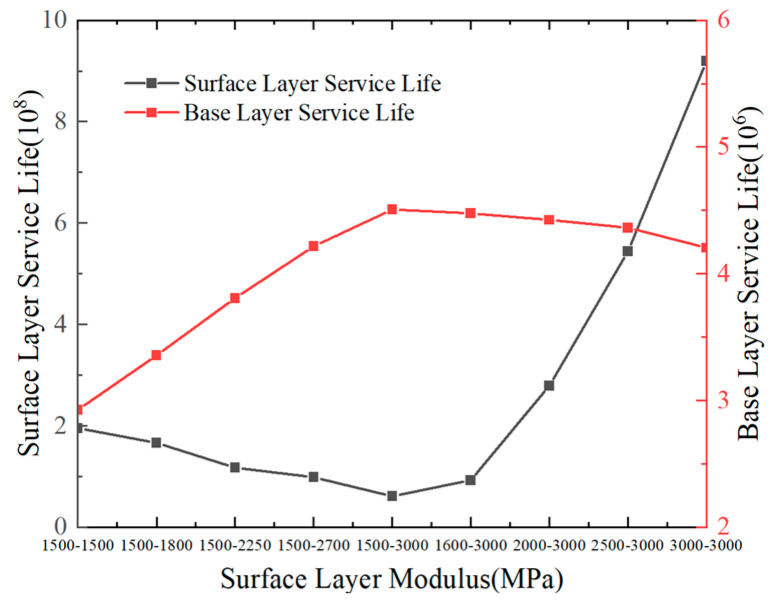
Relationship between fatigue life and surface layer modulus.

Figure 20 shows that the fatigue life of the base course increases with the compressive modulus of the surface layer but decreases with its tensile modulus. A 1500 MPa increase in the compressive modulus results in an improvement of approximately 50% in the base course fatigue life. In contrast, the fatigue life of the surface layer is minimally affected by variations in the compressive modulus but exhibits a dramatic increase with a higher tensile modulus. Specifically, a 1500 MPa increase in the tensile modulus leads to an approximately 14-fold enhancement in the surface layer fatigue life. This analysis demonstrates that the tensile modulus of the asphalt layer exerts a substantial influence on the fatigue life of the surface layer.

**Figure 21:**
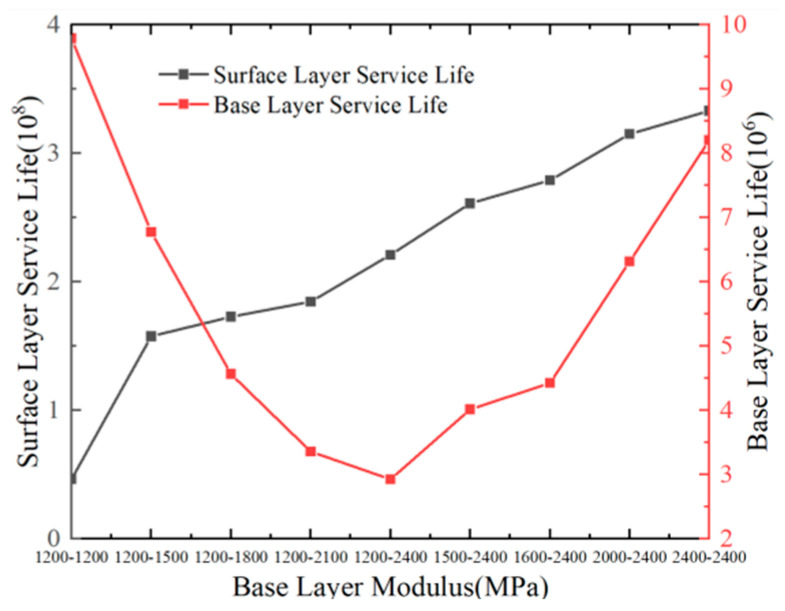
Relationship between fatigue life and base layer modulus.

Figure 21 illustrates that the fatigue life of the pavement surface layer increases with a higher base course modulus. Conversely, the fatigue life of the base course decreases with its compressive modulus but increases with the tensile modulus. A 1200 MPa increase in the base course’s compressive modulus reduces the base course fatigue life by approximately 70%, whereas a 1200 MPa increase in the tensile modulus improves it by approximately 180%. This fact demonstrates that the tensile modulus of the flexible pavement base course has a dominant impact on its fatigue life. To enhance the fatigue life of the base course in pavement structures, it is recommended to reduce the compressive modulus and increase the tensile modulus of the base course.

In conclusion, the fatigue life of the base course is significantly lower than that of the surface layer. To enhance the base course fatigue life, it is recommended to moderately increase the thickness of both the surface layer and base course, reduce the tensile modulus of the surface layer while increasing its compressive modulus, and elevate the tensile modulus of the base course. These measures collectively aim to prolong the service life of the pavement structure.

### 5.2. Optimization Design Analysis of Asphalt Pavement Structure

The multi-factor quantitative model for the tensile and compressive mechanical parameters of pavement materials, established in Section 3.1, demonstrates an accuracy of over 95%. Based on the fatigue life calculations of structural layers presented in Section 4.1, this study recommends moderately increasing the thickness of both the surface and base layers, reducing the tensile modulus while increasing the compressive modulus of the surface layer, and increasing the tensile modulus of the base layer. These adjustments help mitigate the overall mechanical response of the pavement structure, thereby extending its service life. Table 5 presents the proposed pavement structure. Table 6 summarizes the comparison of its mechanical responses and fatigue life with those of a typical asphalt pavement structure.

Table 6 shows that, compared to the conventional pavement structure, the optimized pavement structure exhibits significant reductions in surface transverse tensile strain and maximum horizontal tensile strain at the base course bottom, with reductions of approximately 75% and 20%, respectively. Additionally, the fatigue life of the base course increases by approximately 140%. However, the computational results indicate that the flexible base course asphalt pavement inherently has a lower fatigue life. Based on a design service life of 30 years, Table 7 summarizes the traffic load parameters required to meet this lifespan.

Based on the quantitative models of the mechanical parameters of commonly used pavement materials in Section 1 and the existing research findings [45,46], combined with the mechanical parameters of the optimized asphalt pavement structure in Table 6, the asphalt mixture takes the loading rate of 0.5 MPa/s, the optimum asphalt content, and the temperature of 15 °C under mechanical parameters. The asphalt-treated permeable base (ATB) takes the asphalt content of 3–6% and the temperature of 15 °C under mechanical parameters. Therefore, AC-13 asphalt mixture with 4.5% asphalt content and 3.8% air void content was selected as the upper layer, AC-20 asphalt mixture with 4.1% asphalt content and 4.0% air void content was selected as the middle layer, and AC-25 asphalt mixture with 3.4% asphalt content and 4.2% air void content was selected as the lower layer. The ATB-25 with 3.6~4% asphalt content and 4.8% air voids was selected for the base layer [47]. Table 8 summarizes the detailed mix proportions for the asphalt mixtures and ATB.

## 6. Conclusions

(1) The key mechanical responses of each structural layer in flexible base asphalt pavement showed a good functional relationship with the tensile modulus, compressive modulus, and layer thicknesses. The mix proportion of each structural layer material could be back-calculated by combining the multi-factor quantitative model of tensile and compressive mechanical parameters of pavement materials.

(2) In terms of thickness, the influence of base layer thickness on the key mechanical response of asphalt pavement was more significant than that of surface layer thickness (the maximum influence was more than 40%). In terms of modulus, the influence of the surface layer modulus on the key mechanical response was greater than that of the base layer modulus. The influence of the tensile modulus was more significant than that of compression modulus. Additionally, the maximum influence could reach five times.

(3) The fatigue life of the surface layer was much longer than that of the base layer. The life of the structural layer was more significantly affected by its modulus than by its thickness. Fatigue life could be extended by up to 12 times. The life of the surface layer and the base layer increased with the increment of the thickness of the middle and upper layers and the base layer (the maximum increase is 1.8 times). When designing the structure, the thickness could be appropriately increased, and the tensile modulus could be reasonably controlled.

(4) Based on a large number of structural response analyses, the law of its change with the compression–tension modulus ratio was revealed. Based on this, the optimal compression–tension modulus ratios of the upper and middle surface layers, the lower layer, and the base layer were recommended to be approximately 1.5, 2, and 1.2, respectively. According to the proposed integrated design method, the optimized pavement structure had better stress, the base layer life was significantly extended, and the structural design life was more in line with reality.

This paper conducted a structural analysis based on the concept of integrated design of materials and structures and optimized the structure of flexible base asphalt pavement. The obtained results significantly reduced the mechanical response of each structural layer and significantly increased the fatigue life. However, the research object of this paper was only flexible base asphalt pavement, and comparative research on other types of asphalt pavement structures could be carried out in the future. Furthermore, relying on the test section to conduct actual mechanical response and life analysis improved the material-structure integrated design method established in this paper.

## Figures and Tables

**Table 1:** Mechanical parameter comparison scheme for flexible base asphalt pavement.

Structural Layer Position	Parameter Combination											
	Material	Type	Typical	1	2	3	4	5	6	7	8	9
Upper/Middle Surface Layer	Modified Asphalt Mixture	$E^{t}$	2000	1500	1500	1500	1500	1500	1600	2000	2500	3000
		$u^{t}$	0.2	1500	1800	2250	2700	3000	3000	3000	3000	3000
		$E^{c}$	3000	0.2	0.2	0.2	0.2	0.2	0.2	0.2	0.2	0.24
		$u^{c}$	0.3	0.2	0.24	0.3	0.36	0.4	0.375	0.3	0.24	0.24
		h	10	(8, 10, 12, 14)								
Lower Surface Layer	Matrix Asphalt Mixture	$E^{t}$	1400	1000	1000	1000	1000	1000	1120	1400	1750	2100
		$u^{t}$	0.2	1000	1200	1500	1800	2000	2100	2100	2100	2100
		$E^{c}$	2400	0.2	0.2	0.2	0.2	0.2	0.2	0.2	0.2	0.24
		$u^{c}$	0.3	0.2	0.24	0.3	0.36	0.4	0.375	0.3	0.24	0.24
		h	8	(8, 10, 12, 14)								
Base Layer	Asphalt-Stabilized Aggregate	$E^{t}$	1600	1200	1200	1200	1200	1200	1500	1600	2000	2400
		$u^{t}$	0.2	1200	1500	1800	2100	2400	2400	2400	2400	2400
		$E^{c}$	2400	0.2	0.2	0.2	0.2	0.2	0.2	0.2	0.2	0.24
		$u^{c}$	0.3	0.2	0.25	0.3	0.35	0.4	0.32	0.3	0.24	0.24
		h	30	(28, 32, 34, 36, 38, 40, 44)								
Subbase Layer	Graded Crushed Stone	*E^t^*: 50; *E*^c^: 500; *μ^t^*: 0.035; *μ^c^*: 0.35; *h*: 30 (12, 16, 18, 20, 22, 24, 28)										
Cushion Layer	Graded Crushed Stone	*E^t^*: 40; *E*^c^: 400; *μ^t^*: 0.035; *μ^c^*: 0.35; *h*: 20										
Subgrade	-	*E^t^*: 8; *E*^c^: 80; *μ^t^*: 0.04; *μ^c^*: 0.4										

**Table 2:** Service life prediction parameters for asphalt mixture layers.

Computational Parameters	*β*	*k_b_*	*E_a_* (MPa)	*VFA* (%)	*k_T_*_1_
Value Assignment	1.65	0.9	5000–10,000	70	0.7576

**Table 3:** Service life computational parameters for inorganic binder-stabilized layers.

Computational Parameters	*β*	*k_a_*	*k_T_*_2_	*a*	*b*	*c*_1_	*c*_2_	*c*_3_
Base Course Value Assignment	1.65	0.9	0.7576	13.24	12.52	14	−0.0076	−1.47
Subbase Course Value Assignment	1.65	0.9	0.7576	13.24	12.52	14	−0.0076	−1.47

**Table 4:** The base modulus and the corresponding flexural tensile strength are valued.

Modulus (MPa)	8000–8000	8000–10,000	8000–12,000	8000–15,000	8000–16,000	7500–15,000	10,000–15,000	12,000–15,000	15,000–15,000
Flexural–Tensile Strength (MPa)	1.0	1.2	1.5	1.75	1.8	1.8	1.8	1.8	1.8

**Table 5:** Optimized design of flexible base asphalt pavement structure.

Layer Position	Optimized Pavement Structure					Typical Pavement Structure				
	*h* (cm)	*E*^+^ (MPa)	*μ* ^+^	*E*^−^ (MPa)	*μ* ^−^	*h* (cm)	*E*^+^ (MPa)	*μ* ^+^	*E*^−^ (MPa)	*μ* ^−^
Upper Surface Layer	6	1500	0.2	3000	0.4	4	2000	0.2	3000	0.3
Middle Surface Layer	6	1500	0.2	3000	0.4	6	2000	0.2	3000	0.3
Lower Surface Layer	8	1000	0.2	2000	0.4	8	1400	0.2	2100	0.3
Base Layer	32	2000	0.2	2400	0.24	30	1600	0.2	2400	0.3
Subbase Layer	30	50	0.035	500	0.36	30	50	0.035	500	0.35
Cushion Layer	20	40	0.035	400	0.35	20	40	0.035	400	0.35
Subgrade	-	8	0.04	80	0.4	-	8	0.04	80	0.4

**Table 6:** Comparison of mechanical response and life.

Pavement Structure	Maximum Surface Tensile Strain (10^−6^)	Maximum Base Layer Tensile Stress (MPa)	Maximum Base Layer Tensile Strain (10^−6^)	Surface Layer Service Life (10^8^)	Base Layer Service Life (10^7^)
Optimized Pavement Structure	6.71	0.0978	43.5	0.69	1.02
Typical Pavement Structure	28.03	0.095	54.29	2.36	0.423

**Table 7:** Traffic volume parameters of flexible base asphalt pavement structure.

Initial Year Design Lane Daily Average Single Axle Load Applications N_1_ (times)	Annual Average Growth Rate (%)	Directional Distribution Factor	Lane Coefficient	Design Life (years)
1000	4.5	0.5	0.5	30

**Table 8:** Flexible base asphalt pavement structure and material design.

Layer Position	Optimized Pavement Structure			
	Material Type	Binder Content (%)	Air Voids (%)	Thickness (cm)
Upper Surface Layer	AC-13	4.5	3.8	6
Middle Surface Layer	AC-20	4.1	4	6
Lower Surface Layer	AC-25	3.4	4.2	8
Upper Base Layer	ATB-25	4	4.8	16
Lower Base Layer	ATB-25	3.6	4.8	16

## Data Availability

The raw data supporting the conclusions of this article will be made available by the authors on request.
